# Identification of core gene in chronic rhinosinusitis with nasal polyps and correlations with inflammation-related genes^☆^

**DOI:** 10.1016/j.bjorl.2024.101410

**Published:** 2024-03-01

**Authors:** Jingpu Yang, Chang Liu, Jinzhang Cheng, Yunmeng Wang, Zonggui Wang, Wei Zhong

**Affiliations:** a The Second Hospital of Jilin University, Department of Otolaryngology- Head and Neck Surgery, Changchun, China; b The China-Japan Union Hospital of Jilin University, Department of Ophthalmology, Changchun, China

**Keywords:** Rhinosinusitis, CRSwNP, Bioinformatics, ALOX15

## Abstract

**Objective:**

Our aim in this study is to identify the core genes of chronic rhinosinusitis with nasal polyps and analyze the correlations between it and inflammation-related genes.

**Methods:**

GSE72713 dataset containing gene expression data of ECRSwNP, nonECRSwNP and healthy samples was obtained from Gene Expression Omnibus (GEO) and filtered by limma to identify DEGs among three groups, then the functions and correlated pathways of DEGs were analyzed using GO and KEGG. The core DEGs were selected by the intersection of DEGs and the PPI network was constructed via STRING. The correlations between the expression levels of CRSwNP core gene and inflammation-related genes were analyzed via the Mann-Whitney *U* test.

**Results:**

The DEGs among ECRSwNP, nonECRSwNP, and CTRL were filtered respectively, and enrichment analysis showed they were associated with olfaction and/or immune responses. The PPI network was constructed by 7 core DEGs obtained via the intersection among three groups, and ALOX15 was confirmed as the core gene in the network. Subsequently, the correlations between the expression levels of ALOX15 and inflammation-related genes were illustrated.

**Conclusion:**

In this study, the core gene ALOX15 was selected from the DEGs among ECRSwNP, nonECRSwNP, and CTRL. IL5, IL1RL1, and IL1RAP were found to exhibit a significant positive correlation with ALOX15.

**Level of Evidence:**

Level 3.

## Highlights

• DEGs of CRSwNP were correlated with olfaction and immunization.
• ALOX15 was confirmed as the core gene of CRSwNP.
• IL5, IL1RL1, and IL1RAP were significantly positively correlated with ALOX15.

## Introduction

Rhinosinusitis is a common inflammatory disease that occurs in the mucosa of paranasal sinuses. Based on its duration, it can be classified into Acute Rhinosinusitis (ARS) and Chronic Rhinosinusitis (CRS).^1^ Improper treatment and recurrent episodes often lead to the transformation of ARS into CRS, which can further be categorized as Eosinophilic Chronic Rhinosinusitis (ECRS) or non-Eosinophilic Chronic Rhinosinusitis (non-ECRS) based on the quantitative number of eosinophils in the mucosal tissue.^2^, ^3^ Additionally, in clinical practice, CRS is characterized by two symptoms: the presence of Nasal Polyps (CRSwNP) and the absence of Nasal Polyps (CRSsNP).^4^ The dreadful incidence of CRS significantly impacts the quality of life for millions of individuals and imposes a substantial socioeconomic burden for treatment purposes.^1^, ^5^, ^6^ In the past several years, the understanding of CRS has transcended its previous simplistic conceptualization; yet, a majority of current knowledge remains confined to the phenotypic level, and there still exists an inadequate comprehension of its etiology.^7^ Furthermore, due to its considerable heterogeneity, accurate classification, diagnosis, and treatment according to different subtypes remain challenging. Currently, available knowledge regarding CRS remains insufficient.

In the exploration of any disease, it is crucial to identify the pathogenic or core genes that exhibit abnormal expression patterns during its progression, CRS is no exception. Regrettably, there remains a limited understanding regarding the genetic characteristics underlying CRS. Development in advanced high-throughput sequencing technology has facilitated a deeper exploration of gene expression patterns during the pathological processes associated with CRS.^7^ In recent years, extensive researches utilizing DNA microarray technology have been conducted to explore the gene expression of CRSwNP,^8^, ^9^, ^10^, ^11^ yielding promising results that contribute to unraveling the highly intricate pathogenic mechanisms and pathways of CRSwNP. However, the accurate identification of biomarkers for Eosinophilic Chronic Rhinosinusitis with Nasal Polyps (ECRSwNP) and non-Eosinophilic Chronic Rhinosinusitis with Nasal Polyps (nonECRSwNP) remains elusive.^12^ Therefore, it is evident that researchers should allocate more efforts towards discovering valuable genetic markers specific to each subtype of CRS so as to more comprehensively reveal the pathogenesis of each subtype of CRS and precisely distinguish each subtype at the level of gene expression. This will outstandingly contribute to the discovery of potential drug therapy targets and the realization of accurate treatment for diverse subtypes of CRS. Additionally, it has been reported that the inflammatory process of CRSwNP is mainly mediated by the Th2 cell immune response, of which IL-33 plays a critical role in the development and regulation.^13^, ^14^ However, to our knowledge, there remains a dearth of information regarding the interplay between core genes associated with CRS and the genes involved in the IL-33/ST2 signaling pathway.

Our objective in this research was to identify core genes that potentially contribute to the pathogenesis of ECRSwNP and nonECRSwNP utilizing bioinformatics techniques, followed by experimental validation using qRT-PCR technology, while also investigating their interactions with inflammation-related genes and crucial genes in the IL33/ST2 pathway. Furthermore, we aimed to explore the expression patterns of these genes across different subtypes of CRSwNP. We anticipated that the identified core gene in the current study held promise as potential biomarkers for distinguishing ECRSwNP from nonECRSwNP and would offer prospective targets for novel therapeutic drugs in CRSwNP, thereby providing valuable insights and inspirations to advance precise treatment strategies of CRSwNP.

## Methods

### Data source

In this research, GSE72713 dataset (platform: GPL11154 Illumina HiSeq 2000 (Homo sapiens) which contains sequencing data of rhinosinusitis samples was acquired from the Gene Expression Omnibus (GEO) database.^11^ (https://gdc-portal.nci.nih.gov/). There were a total of 9 samples in the original expression matrix, including 3 samples of Eosinophilic Chronic Rhinosinusitis with Nasal Polyps (ECRSwNP), 3 samples of non-Eosinophilic Chronic Rhinosinusitis with Nasal Polyps (nonECRSwNP), and 3 samples of normal tissue (CTRL).

### Differentially expressed genes analysis

The limma (version 1.34.0)^15^ was employed to perform differential analysis on the expression matrix of sequencing data in all chips to identify Differentially Expressed Genes (DEGs) among ECRSwNP, nonECRSwNP, and CTRL. We set |logFC| > 1 and adjusted *p*-value < 0.05 as the threshold. Thereafter, the DEGs were obtained and used for subsequent analysis.

### Function enrichment analysis

The DEGs identified from the GSE72713 dataset in all three groups were conducted Gene Ontology (GO) and Kyoto Encyclopedia of Genes and Genomes (KEGG) pathways annotation analysis using the R package clusterProfiler (version 4.2.2).^16^ The *p*.value corrected by Benjamini-Hochberg (BH) less than 0.05 and FDR value (q.value) < 0.05 were considered statistically significant. The top 10 pathways exhibiting the smallest *p-*value (i.e., the most statistically significant pathways enriched by DEGs) were subsequently selected and visualized.

### Protein-protein interaction (PPI) network establishment

In this research, the intersection of three DEG lists among ECRSwNP, nonECRSwNP, and CTRL was obtained resulting in a total of 236 intersecting DEGs. Subsequently, all DEGs were input into the STRING database^16^ (https://cn.string-db.org/) for establishing a PPI network. The PPI data file, which was obtained using default parameters in all analyses and excluded independent DEGs without any interactions, was imported into Cytoscape software^17^ for visualization.

### qRT-PCR

Total RNA was extracted from nasal polyp tissue samples using the TRIzol method, followed by reverse transcription to obtain cDNA. The reaction system was constructed using TransScript® II First-Strand cDNA Synthesis SuperMix (TransGen Biotech), then mixed and centrifuged, followed by incubation at 50 °C for 15 min and subsequently exposed to a temperature of 85 °C for 5 s to deactivate the enzyme. The product was placed on ice immediately after the reaction. Subsequently, qRT-PCR experiments were performed to validate the mRNA expression levels of ALOX15, EMR1, CCL24, CCL13, and PLAT. The reaction system was constructed using ChamQ Universal SYBR qPCR master Mix (Vazyme Biotech), then the amplification reaction was performed with reaction conditions as follows: 1 cycle of 90 °C for 30 s, 40 cycles of 95 °C for 10 s and 60 °C for 30 s. The primer sequences can be found in Supplemental Table 1. The relative expression levels of each gene were calculated using the 2^−ΔΔCT^ method with GAPDH as a reference for normalization.

### Statistical analysis

Bioinformatics data processing and analysis were executed using R software (version 4.1.1). For comparisons between two groups of continuous variables, the statistical significance of normal distribution variables was assessed using the Independent Student t-test, while discrepancies in non-normal distribution variables were analyzed using the Mann-Whitney *U* test (Wilcoxon rank-sum test). The correlation analysis of gene expression levels was performed using the Pearson correlation coefficient and adjusted by the False Discovery Rate (FDR) method. The results of qRT-PCR were analyzed and visualized using GraphPad Prism 9. Group comparisons were performed using the one-way analysis of variance (ANOVA), followed by Tukey's post-hoc test. All *p-*values were two-sided, with *p* < 0.05 being considered statistically significant.

## Results

### Identification of DEGs among ECRSwNP, nonECRSwNP, and CTRL groups

**Figure 1:**
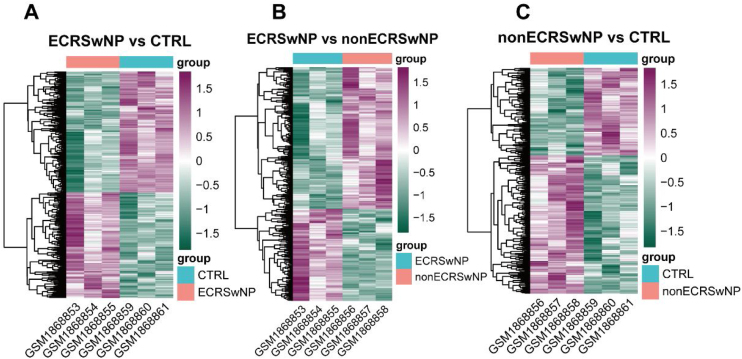
Heatmap of DEG expression levels among the three groups. (A) Heatmap of DEGs between ECRSwNP and CTRL. (B) Heatmap of DEGs between ECRSwNP and nonECRSwNP. (C) Heatmap of DEGs between nonECRSwNP and CTRL. Purple indicates high expression, while green indicates low expression. DEG, Differentially Expressed Genes; ECRSwNP, Eosinophilic Chronic Rhinosinusitis with Nasal Polyps; nonECRSwNP, non-Eosinophilic Chronic Rhinosinusitis with Nasal Polyps; CTRL, Control.

In the first instance, the differential analysis of gene expression among samples in ECRSwNP, nonECRSwNP, and CTRL groups was implemented in the current study. The results exhibited that a total of 952 DEGs were identified between the ECRSwNP and CTRL groups, of which 444 DEGs were significantly up regulated in ECRSwNP (Fig. 1A). Whereas there were 424 DEGs detected between ECRSwNP and nonECRSwNP groups, with 167 DEGs significantly up-regulated in ECRSwNP and 257 DEGs significantly up-regulated in nonECRSwNP (Fig. 1B). Furthermore, we discovered a total of 472 DEGs between nonECRSwNP and CTRL groups, containing 289/183 DEGs with significantly increased expression in the nonECRSwNP group and the CTRL group, respectively (Fig. 1C).

### Enrichment analysis of DEGs

**Figure 2:**
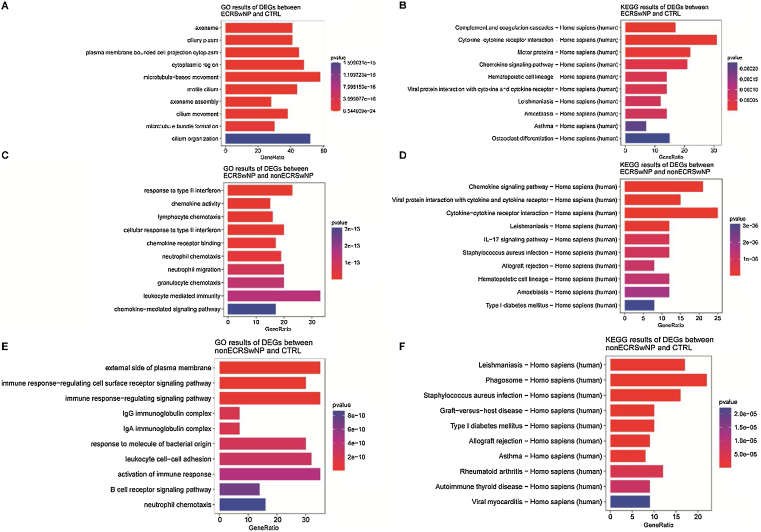
Enrichment analysis of DEGs between ECRSwNP, nonECRSwNP, and CTRL. (A) GO enrichment analysis of DEGs between ECRSwNP and CTRL. (B) KEGG enrichment analysis of DEGs between ECRSwNP and CTRL. (C) GO enrichment analysis of DEGs between ECRSwNP and nonECRSwNP. (D) KEGG enrichment analysis of DEGs between ECRSwNP and nonECRSwNP. (E) GO enrichment analysis of DEGs between nonECRSwNP and CTRL. (F) KEGG enrichment analysis of DEGs between nonECRSwNP and CTRL. The length of the cylinder indicates the number of enriched DEGs in a pathway, and the red color of the cylinder indicates the higher level of significance (smaller *p-*value). DEG, Differentially Expressed Genes; ECRSwNP, Eosinophilic Chronic Rhinosinusitis with Nasal Polyps; nonECRSwNP, non-Eosinophilic Chronic Rhinosinusitis with Nasal Polyps; CTRL, Control.

Subsequently, with the aim of better understanding the role of DEGs in the pathways and the biological mechanism of DEGs affecting the occurrence and development of rhinosinusitis, KEGG, and GO enrichment analyses were carried out for all DEGs. GO enrichment analysis revealed a significant enrichment of the DEGs between the ECRSwNP and CTRL in biological terms associated with olfaction, such as motile cilium and microtubule-based movement (Fig. 2A). The result of KEGG enrichment analysis indicated the above DEGs were significantly enriched in Asthma and some pathways related to protein processing (Fig. 2B).

Meanwhile, the enrichment analysis was carried out for the DEGs between the ECRSwNP and nonECRSwNP likewise. Interestingly, the results of GO and KEGG analysis demonstrated the prominent terms and pathways were mostly associated with immune response, such as response to type II interferon and chemokine activity (Fig. 2C), Chemokine signaling pathway and IL-17 signaling pathway (Fig. 2D).

We eventually obtained the enrichment analysis results of the DEGs between the nonECRSwNP and CTRL as well. As exhibited in Fig. 2E, these genes were enriched in the pathways of immunization, to name only a few, immune response-regulating signaling pathway and neutrophil chemotaxis by GO analysis. Conversely, KEGG analysis revealed the involved pathways were correlated with both olfaction and immunization (Fig. 2F).

### PPI network of core DEGs

**Figure 3:**
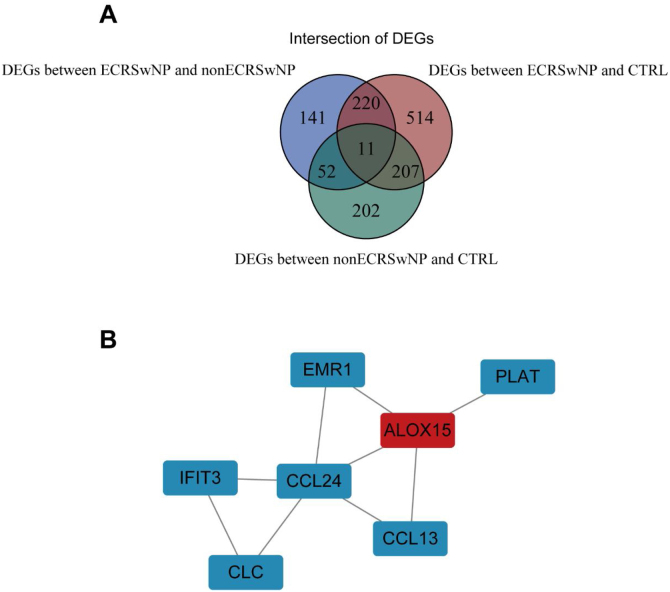
Construction of the PPI network for DEGs. (A) A total of 11 intersection genes in DEGs between the three groups. (B) PPI network constructed by 7 core DEGs.

So as to further explore the interaction between DEGs, we first screened out the intersection of DEGs among the three groups, namely core DEGs, which may play a pivotal role in the occurrence and development of rhinosinusitis. A total of 11 core DEGs were acquired (Fig. 3A). A PPI network was constructed comprising 7 DEGs with intricate gene interactions and numerous DEGs (Fig. 3B). The network suggested the core DEGs may exert diverse interactions, thereby influencing the progression of rhinosinusitis.

### Correlation of the expression levels between ALOX15 and key genes

**Figure 4:**
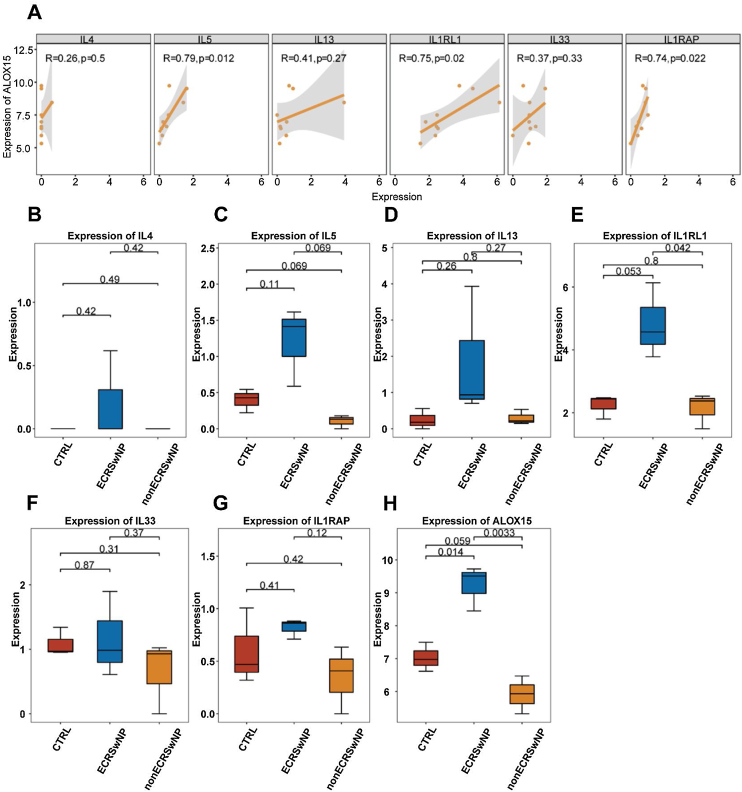
Correlation between the expression levels of ALOX15 and inflammation-related key genes (A) Correlation between expression levels calculated by Pearson coefficient, with *p* < 0.05 considered to be significant. (B) Box plot of the difference in expression levels of IL4. (C) Box plot of the difference in expression levels of IL5. (D) Box plot of the difference in expression levels of IL13. (E) Box plot of the difference in expression levels of IL1RL1. (F) Box plot of the difference in expression levels of IL33. (G) Box plot of the difference in expression levels of IL1RAP. (H) Box plot of the difference in expression levels of ALOX15.

The pathogenesis and pathological progression of CRSwNP have been found to be intricately associated with the inflammatory process and pathways. Thus, for the sake of elucidating the correlations between ALOX15 and expression levels of inflammatory factors IL-4, IL-5, and IL-13, as well as the key genes in the IL33/ST2 pathway including IL1RL1, IL33, and IL1RAP, the correlations of the expression levels of the above genes were analyzed in the present research. As depicted in Fig. 4A, the expression levels of IL5 (*p* = 0.012), IL1RL1 (*p* = 0.02), and IL1RAP (*p* = 0.022) were significantly positively correlated with ALOX15, suggesting potential synergistic effects among these genes. However, no significant correlations were observed between ALOX15 expression and the expression levels of IL4, IL13, and IL33.

Besides, the expression levels of the above seven genes in different CRS subtypes were analyzed as well. The results exhibited no significant differences in the expression levels of IL4 (Fig. 4B), IL5 (Fig. 4C), IL13 (Fig. 4D), IL33 (Fig. 4F), and IL1RAP (Fig. 4G) among the three groups. In comparison, the expression level of IL1RL1 was significantly higher in ECRSwNP compared to nonECRSwNP (Fig. 4E). Additionally, ALOX15 exhibited a significantly higher expression level in ECRSwNP than in both nonECRSwNP and CTRL (Fig. 4H).

### qRT-PCR validation

**Figure 5:**
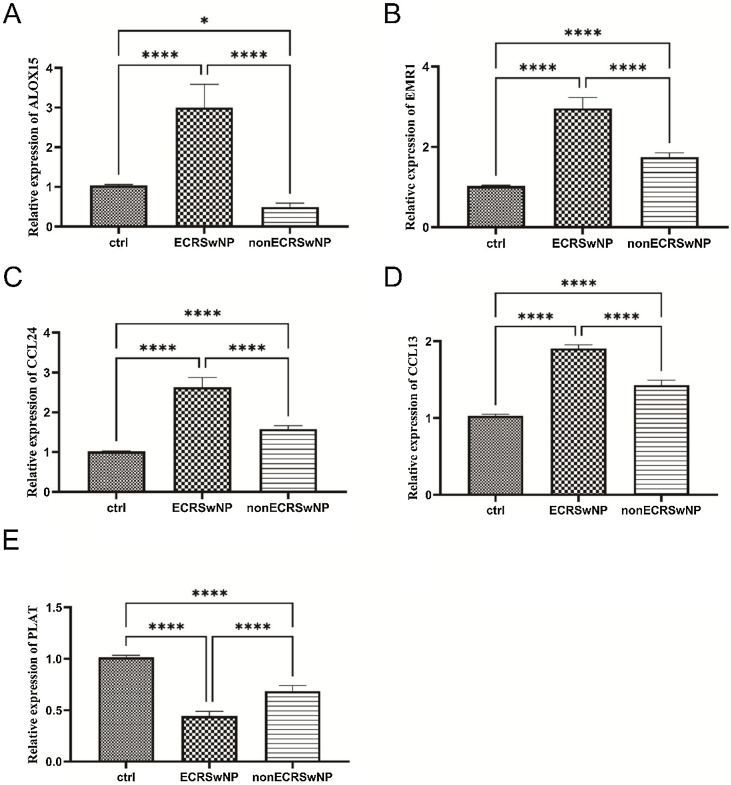
The mRNA expression levels validation of ECRSwNP and nonECRSwNP by qRT-PCR. (A) The relative mRNA expression of ALOX15. (B) The relative mRNA expression of EMR1. (C) The relative mRNA expression of CCL24. (D) The relative mRNA expression of CCL13. (E) The relative mRNA expression of PLAT. *p < 0.05; ****p < 0.0001.

Finally, qRT-PCR was employed to assess the mRNA expression levels of the above-mentioned core gene ALOX15, eosinophil-specific receptor gene EMR1, cytokine CC gene family CCL24, CCL13, and plasminogen activator-encoding gene PLAT. As depicted in Fig. 5A, compared to the control group, a significant upregulation of ALOX15 mRNA expression was observed in the ECRSwNP group while a notable downregulation was evident in the nonECRSwNP group, consistent with the findings from bioinformatics analysis. The qRT-PCR results for EMR1, CCL24, and CCL13 genes demonstrated that their mRNA expressions were significantly elevated in both ECRSwNP and nonECRSwNP groups when compared to the control group; moreover, these expressions were highest in the ECRSwNP group (Fig. 5B‒5 D). Conversely, PLAT exhibited an opposite trend to these three genes; its expression level decreased significantly in both ECRSwNP and nonECRSwNP groups compared to the control group with the lowest expression observed in the ECRSwNP group (Fig. 5E).

## Discussion

The impact of CRS on the quality of life for millions of patients worldwide is profound. Owing to its extremely intricate heterogeneity, at present, most treatment approaches primarily address symptoms, leading to a high risk of relapse and imposing significant psychological and economic burdens on patients.^5^ The two subtypes of CRSwNP, ECRSwNP, and nonECRSwNP, exhibit distinct inflammatory characteristics, resulting in disparate symptomatology and prognostic profiles.^11^, ^18^ However, the current understanding of these subtypes and their genetic mechanisms at the molecular level remains insufficiently aligned, posing significant challenges to accurate diagnosis and treatment. In the current study, we employed bioinformatics technology to screen out DEGs between patients with ECRSwNP, non-ECRSwNP patients, and healthy controls. Additionally, we conducted functional enrichment analysis on these DEGs, whose results exhibited enrichment of immune and/or olfactory-related pathways. Subsequently, we constructed a PPI network centered around ALOX15, which included 7 core DEGs exhibiting strong interactions with ALOX15. Additionally, we explored the correlation between ALOX15 and the expression levels of inflammatory factors IL-4, IL-5, and IL-13, as well as important genes in the IL33/ST2 pathway including IL1RL1, IL33, and IL1RAP. The results revealed a significant positive correlation between ALOX15 and the expression levels of IL5, IL1RL1, and IL1RAP. Since elevated levels of Th2 cell marker IL-5 were found to be associated with CRS occurrence and development in previous studies, this result further supports that CRS is linked to inflammatory response processes involving Th2 cells.^20^, ^21^ Finally, we investigated the expression levels of these 7 genes among different subtypes of CRSwNP. Our findings indicated the expression level of IL1RL1 was significantly higher in ECRSwNP compared to nonECRSwNP. Similarly, the expression level of ALOX15 was significantly higher in ECRSwNP compared to both nonECRSwNP and healthy controls.

The core gene identified in the current study is ALOX15, whose full name is arachidonate 15-lipoxygenase, mainly responsible for encoding a member of the protein-lipid oxygenase family, 15-Lipoxygenase (15-LOX) type 1. As a highly conservative member of the family, this enzyme mediates the enzymatic oxidation of polyunsaturated fatty acids, thereby facilitating the production of diverse bioactive lipid mediators.^19^ It has been demonstrated that 15-LOX played a role in the pathogenesis of various chronic inflammatory diseases,^20^ while also exhibiting certain anti-inflammatory effects.^21^ Kelavkar et al.^22^ reported an up-regulation of 15-LOX in Nasal Polyps (NP) tissues. In several recent researches, Wang et al.^23^ revealed the involvement of ALOX15^+^ macrophages in type 2 immune-driven pathogenesis of ECRS and NP through chemokine secretion to recruit eosinophils, monocytes, and Th2 cells. Kristjansson, et al.^24^ exhibited that loss-of-function variants of the ALOX15 gene had a protective effect on both NP and CRS, suggesting 15-LOX as a potential therapeutic target for these conditions. Yoshimasa et al.,^25^ on the other hand, highlighted the significance of 15-LOX-1 as a key molecule promoting eosinophilic inflammation in ECRS. Noteworthily, Liang et al.^26^ quantified the expression level of ALOX15 mRNA in polyp tissues obtained from 48 patients with CRSwNP using qRT-PCR and other techniques. They observed a significantly higher ALOX15 mRNA level in patients with ECRSwNP compared to nonECRSwNP patients, and preliminarily judged the potential role for ALOX15 as a predictive marker for ECRSwNP. The findings of the current study were consistent with previous research, providing further evidence for the significant role of the ALOX15 gene in CRS pathogenesis. These results further confirmed that ALOX15 may serve as a valuable biomarker and potential therapeutic target for CRS treatment.

There are certain limitations in the current study. Firstly, further validation of ALOX15 as a reliable biomarker for ECRSwNP is required through comprehensive expression level analysis in other large-scale CRS datasets and clinical information verification in future investigations. Secondly, the investigation on nonECRSwNP remains insufficient compared to ECRSwNP. Despite our efforts, we were unable to identify a potential gene marker for nonECRSwNP. Thirdly, in conjunction with prior investigations, our findings provided further substantiation and validation of the pivotal role of the ALOX15 gene in ECRSwNP. However, no novel potential biomarkers for ECRSwNP were identified, thereby rendering our work marginally less groundbreaking but more closely aligned with clinical applicability. Although this research has some shortcomings, it provides valuable insights into unraveling the pathogenic mechanism of CRSwNP at the genetic level and achieving accurate differentiation of CRSwNP subtypes.

## Conclusions

In conclusion, the core DEG associated with CRSwNP, namely ALOX15, was selected from the DEGs among ECRSwNP, nonECRSwNP, and healthy control. The positive correlations of the expression between ALOX15 and IL5, IL1RL1, and IL1RAP were confirmed and the significantly higher expression of ALOX15 was found in ECRSwNP. In brief, ALOX15 is a potential biomarker of CRSwNP subtype identification.

## Ethics approval and consent to participate

Not applicable.

## Consent for publication

Not applicable.

## Data availability statement

The datasets used and/or analyzed during the current study are available from the corresponding author upon reasonable request.

## Author contributions

Jingpu Yang: Writing – original draft, writing – review & editing. Chang Liu and Jinzhang Cheng: Data curation, Methodology. Yunmeng Wang: Validation. Zonggui Wang and Wei Zhong: Conceptualization, Writing – review & editing. All authors have read and approved the final manuscript.

## Funding

This study was supported by grants from the Science and Technology Department of Jilin Province (nº 20200201517JC, nº 20220203114SF).

## Conflicts of interest

The authors declare no conflicts of interest.
