# Supplementary material for: Identification of core gene in chronic rhinosinusitis with nasal polyps and correlations with inflammation-related genes
Source: Braz J Otorhinolaryngol. 2024 Mar 1;90(3):101410. doi: 10.1016/j.bjorl.2024.101410 (PMC10955304; doi:10.1016/j.bjorl.2024.101410)
Supplement: Supplementary file 1 [file mmc1.docx]

BJORL-D-23-00270_Supplementary Material

**Supplemental** **Table 1** Primer sequences.

| **Genes** | **Primer direction** | **Sequences** |
| --- | --- | --- |
| ALOX15 | F | CTCAAGGTGGAAGTACCGGAGTA |
|  | R | TGCCGTTTGCGCAGTTT |
| EMR1 | F | TGGAGGCTGTGATACTGTTCTTGA |
| CCL24 | R | CTTGATGTTGCGAGAGCTGAAG |
|  | F | GCAGGCCTGATGACCATAGTAAC |
|  | R | GATGATGTGGTGGGCACAGA |
| CCL13 | F | TGCTCATGACAGCAGCTTTCA |
|  | R | ATGGGACGTTGAGTGCATCTG |
| PLAT | F | GCGAGCCAAGGTGTTTCAA |
|  | R | CTGGCACACGAAATCTGAGAAG |
| GAPDH | F | GAAGGTGAAGGTCGGAGTC |
|  | R | GAAGATGGTGATGGGATTTC |
